# Effect of Stirrups on Plate End Debonding in Reinforced Concrete Beams Strengthened with Fiber Reinforced Polymers

**DOI:** 10.3390/polym13193322

**Published:** 2021-09-28

**Authors:** Abdulaziz I. Al-Negheimish, Ahmed K. El-Sayed, Mohammed A. Al-Saawani, Abdulrahman M. Alhozaimy

**Affiliations:** Center of Excellence for Concrete Research and Testing, Department of Civil Engineering, College of Engineering, King Saud University, P.O. Box 800, Riyadh 11421, Saudi Arabia; negaimsh@ksu.edu.sa (A.I.A.-N.); malsaawani@ksu.edu.sa (M.A.A.-S.); alhozimy@ksu.edu.sa (A.M.A.)

**Keywords:** concrete beams, FRP strengthening, plate end debonding, stirrups

## Abstract

Plate end (PE) debonding is one of the critical debonding failure modes that may occur in reinforced concrete (RC) beams strengthened with externally bonded fiber reinforced polymers (FRPs). This study investigated the effect of internal steel stirrups on the PE debonding failure load of FRP-strengthened RC beams. The dimensions of the beams were 3400 × 400 × 200 mm. The beams were strengthened with carbon FRP (CFRP) sheets bonded to the soffit of the beams. The beams were divided into two series based on the distance of the cutoff point of the CFRP sheets from the nearest support. This distance was 50 mm or 300 mm, and the amount of steel stirrups was varied in terms of varying the stirrup diameter and spacing. The beams were simply supported and tested under four-point bending. The test results indicate that the effect of stirrups on the load carrying capacity of the beams was more pronounced for the beams with CFRP sheets extended close to the supports. It was also indicated that beams with larger amounts of stirrups failed in PE debonding by concrete cover separation while beams with lower amounts of stirrups failed in PE by either PE interfacial debonding or critical diagonal crack-induced debonding. The beams were analyzed using several analytical models from design guidelines and the literature. The result of analysis indicates that most of the available models failed to reflect the effect of stirrups in predicting PE debonding failure load of the beams. On the other hand, the models of El-Sayed et al. and Teng and Yao were able to capture such an effect with the best predictions provided by El-Sayed et al. model.

## 1. Introduction

Fiber reinforced polymers (FRPs) are widely used in repair and strengthening applications of reinforced concrete (RC) and masonry structures [1,2,3,4,5]. They have proven to provide a suitable solution for strengthening such structures and are considered a more attractive alternative over traditional strengthening techniques. This is mainly because of their excellent characteristics, such as high strength-to-weight ratio, corrosion resistance, ease of installation, minimal change in structural dimensions, and aesthetic appearance. Flexural strengthening of RC beams by external bonding of FRP sheets or plates to the tension face of the beams is one of the common strengthening practices. However, a fraction of the strain capacity of the FRP is utilized, as premature debonding of FRP from the concrete substrate may occur. Debonding of FRP generally initiates in regions of high stress concentration at the FRP–concrete interface. There are two main debonding failure modes: intermediate crack (IC)-induced debonding and plate end (PE) debonding. IC debonding occurs in FRP-strengthened RC beams in the region of high moment due to the opening of flexural cracks [6,7,8,9,10,11,12,13]. On the other hand, PE debonding initiates at the plate end region due to high interfacial and normal stresses [14,15,16,17,18,19,20,21]. The utilization of strain capacity of FRP is generally low in strengthened beams failing by PE debonding compared with those failing by IC debonding. A recent study [6] on carbon FRP (CFRP) strengthened RC beams tested under different shear span to depth ratios has indicated a CFRP strain range of 12.9% to 27.2% of the CFRP rupture strain at PE debonding. Meanwhile, the corresponding range at IC debonding was 33.3% to 40.1% of the CFRP rupture strain. The lower efficiency of FRP-strengthening system in beams experiencing PE debonding combined with the brittle nature of this failure make it a more critical mode of failure. Therefore, anchorage of the FRP plates at their ends is a logical method for improving the efficiency of flexural FRP-strengthening by preventing or delaying PE debonding failure. Several studies have been conducted to investigate the effect of installing U-shaped FRP jackets at the plate ends [1,22,23,24]. The findings of these studies indicated that more utilization of FRP strain capacity of the flexural strengthening can be realized by the installation of FRP U jackets. In fact, the use of FRP U jackets not only serves to delay or prevent PE debonding but also serves to increase the shear capacity of the beams. Generally, flexural strengthening of RC beams using FRP laminates requires that the shear strength of the beam be checked. This is to ensure that no shear failure occurs before reaching the improved flexural capacity of the beam. Several studies indicated that the shear capacity of the beams was increased by using FRP U jackets [25,26,27].

Different studies on PE debonding in FRP-strengthened RC beams indicated the relationship between the occurrence of such a mode of failure and the presence of shear cracks at the plate end region [6,28,29,30,31]. Garden and Hollaway [28] and Garden et al. [29] indicated that the formation and widening of the shear and tributary cracks at the plate end region were responsible for the initiation of PE debonding failure. A similar observation was also reported by Smith and Teng [30], Pham and Al-Mahaidi [31], and Al-Saawani et al. [6], where PE debonding took place after the formation of shear cracks at the end of FRP plates. Some studies also revealed the relationship between PE debonding failure load and nominal shear capacity of FRP-strengthened RC beams. The work of Buyukozturk et al. [32] showed the increase in load carrying capacity with the increase in nominal shear capacity of beams suffering from PE debonding. Li et al. [33] also indicated the effect of nominal shear resistance of FRP-strengthened RC beams on the debonding load carrying capacity of the beams. In fact, there are many existing models for predicting the PE debonding failure load which were developed by different researchers based on the shear capacity of the beams [14,15,19,21,34,35,36,37]. Also, the design guidelines of fib Bulletin 90 [38], ACI 440.2R-17 [39], AS 5100.8 [40] and Concrete Society Technical Report 55 [41], provide shear-based models for evaluating PE debonding failure load in FRP-strengthened RC beams.

El-Sayed et al. [14] established a database of 128 beam test results that failed in PE debonding. They found that at failure, the shear forces at the plate end ranged from 0.98 to 2.91 of the concrete shear cracking load *V_c_* calculated by ACI 318-19 code [42]. This wide range is attributed to the different parameters that influence PE debonding. One of these parameters is the amount of internal stirrups that resist shear stresses. The existence of the stirrups at the FRP plate end region restricts the widening of the shear cracks in this region that are responsible for the initiation of PE debonding failure. Shear cracks are generally associated with relative horizontal and vertical displacements between the two surfaces of the crack. These horizontal and vertical displacements increase the interfacial and normal stresses, respectively. Nevertheless, no systematic study has been carried out to investigate the effect of stirrups on PE debonding. Therefore, the research described in this paper was directed to investigate such an effect to bridge the gap in this area.

### 1.1. PE Debonding Failure

**Figure 1:**
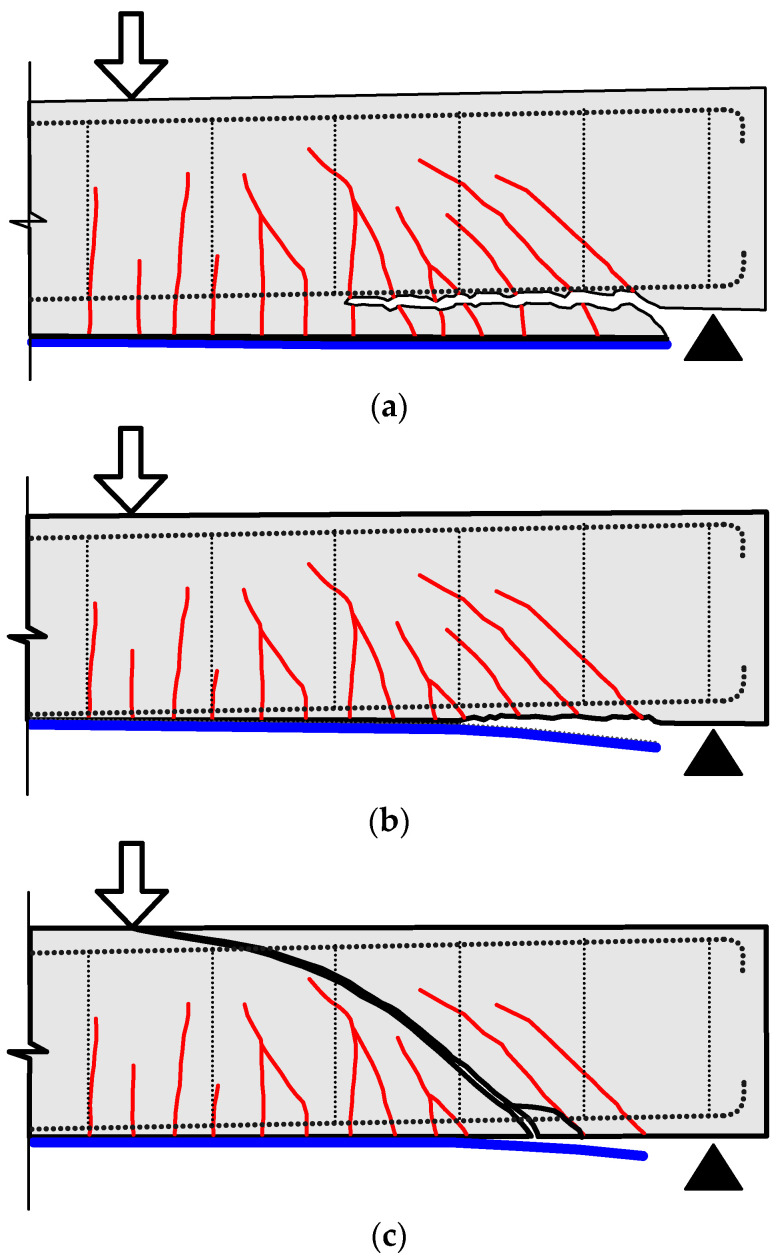
Plate end debonding modes: (**a**) Concrete cover separation; (**b**) Plate end interfacial debonding; (**c**) Critical diagonal crack debonding.

PE debonding may occur in three different modes: concrete cover separation (CCS), PE interfacial debonding, and critical diagonal crack (CDC)-induced debonding [20]. Figure 1 shows schematically the three types of PE debonding failure. CCS occurs by separation of the concrete cover at the level of the main tension steel, as shown in Figure 1a. It is considered the most common PE debonding failure, which initiates at the plate end and propagates towards the middle of the beam. PE interfacial (PEI) debonding also initiates at a plate end and propagates along the interface of concrete substrate and FRP without reaching the level of tension steel, as shown in Figure 1b. CDC debonding takes place due to the formation of major shear crack that extends to the bottom of the beam intersecting the FRP plate near its end. The debonding starts at the intersection point propagating to the plate end along the concrete–FRP interface [20], as shown in Figure 1c. CDC debonding generally occurs in beams with a lower amount of shear reinforcement. On the other hand, for a strengthened beam failing in PE debonding, one cannot confirm that the beam will fail in CCS or PE interfacial debonding [17]. This is because the two modes of failure initiate at the plate end and propagate towards the center of the beam, and they are related to the same cause of high interfacial and normal stresses near the plate end [18].

### 1.2. Research Significance

PE debonding is a critical mode of failure. Experimental observations indicated the occurrence of such a mode of failure after the formation of shear cracks at the plate end region. Since the existence of the reinforcing stirrups controls the opening of shear cracks, it is anticipated that the stirrups may also affect the PE debonding capacity of the beams. The present experimental study was conducted to evaluate the PE debonding failure load as affected by the amount of steel stirrups in RC beams strengthened with CFRP composites. The study provides experimental data considering the effect of stirrup diameter and spacing. Furthermore, the beams were analyzed using the available PE debonding design models. The results of this analysis are also presented and discussed. The findings of this study increase the background knowledge on the PE debonding failure and help validate or improve the relevant design provisions currently in effect.

## 2. Experimental Investigation

The experimental program described in this paper consisted of flexural tests conducted on full scale RC beams strengthened with externally bonded CFRP composites. The tests were carried out on beams strengthened with CFRP sheets extended close to the supports or terminated in the shear span away from the supports.

### 2.1. Test Beams

**Figure 2:**
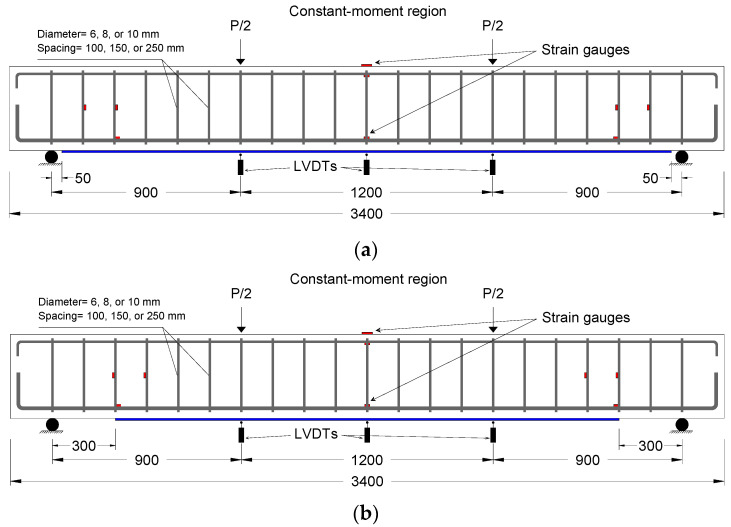

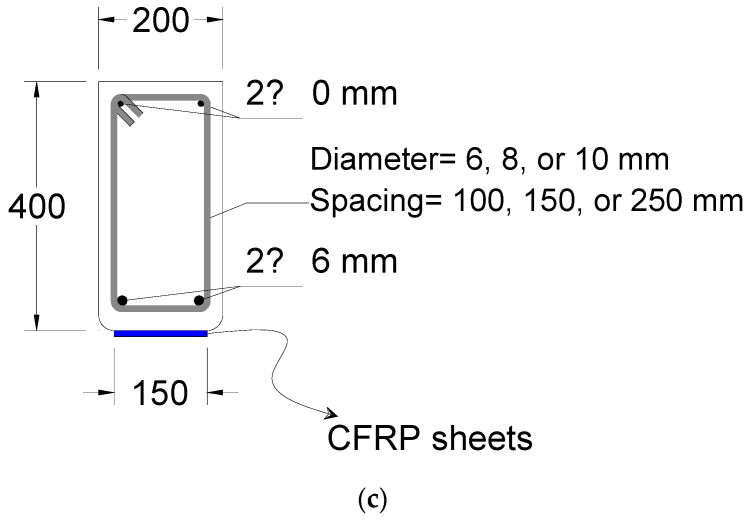
Details of test beams: (**a**) Beams of Series 1; (**b**) Beams of Series 2; (**c**) Cross-section. (All dimensions in mm)

The experimental program included 10 full-scale RC beams that were constructed and tested up to failure. The beams had a total length of 3400 mm, an overall depth of 400 mm, and a width of 200 mm, as presented in Figure 2. The beams were strengthened in flexure using external CFRP sheets bonded to the tension face of the beams. The beams were divided into two series according to the distance of the termination point of the CFRP sheets to the nearest support. Each series included five beams. Series 1 had the CFRP sheets extended close to the supports with an unplated length, L_up_, of 50 mm measured from the cutoff point of the sheets to the support, as shown in Figure 2a. On the other hand, the beams of Series 2 had the distance of the cutoff point of the sheets to the support of 300 mm, as shown in Figure 2b. The width of CFRP sheets was maintained at 150 mm for all beams. The variables in each series were the diameter and spacing of the stirrups. Three different stirrup diameters of 6, 8, and 10 mm were used. Likewise, three different stirrup spacings of 100, 150, and 250 mm were used. These variations in stirrup diameter and spacing yielded a range of stirrup reinforcement ratios from 0.20% to 0.79%. All beams were reinforced with two steel bars of 16 mm diameter as main tensile reinforcement and two steel bars of 10 mm diameter as top reinforcement, as shown in Figure 2. Table 1 summarizes the test variables of the beams. The designation of the beams consisted of three parts. The first part stands for the stirrup diameter, whereas the second part refers to the stirrup spacing. The third part stands for the distance of the cutoff point of the CFRP sheets to the support.

### 2.2. Materials

The tested beams were constructed using a ready-mix concrete provided by a local supplier. The concrete strength was determined by testing three standard concrete cylinders of 150 mm diameter and 300 mm height. The cylinders were prepared using the same concrete used in the beams and were cured under the same conditions of the beams. The cylinders were tested at the time of beam testing following ASTM C39/C39M-18 standard [43] and gave an average concrete compressive strength of 35 MPa with a coefficient of variation of 4.0%.

The steel bars used in reinforcing the beams had a deformed surface except for 6 mm diameter bars, which had a smooth surface. The tensile properties of the bars were determined by testing three samples of each bar diameter according to ASTM A370-19e1 standard [44]. These properties are given in Table 2.

A wet layup system, composed of carbon fabric sheets and adhesive, was used in strengthening the test beams. The sheets were made of unidirectional carbon fibers and were commercially known as Tyfo-SCH-41 (FYFE, San Diego, CA, USA). The sheets had an equivalent thickness of 0.41 mm and a weight of 644 gm per square meter. The adhesive was a two-component epoxy matrix commercially known as Tyfo S Epoxy (FYFE, San Diego, CA, USA). The tensile properties of the carbon fabric and adhesive are given in Table 2, as provided by the manufacturer.

### 2.3. Strengthening Procedure

**Figure 3:**
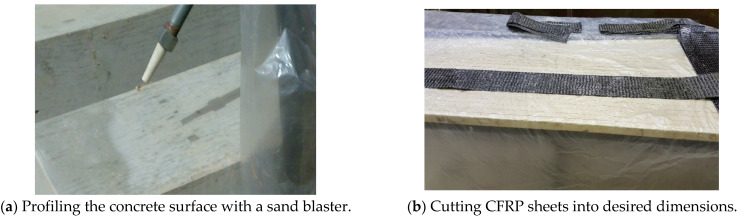

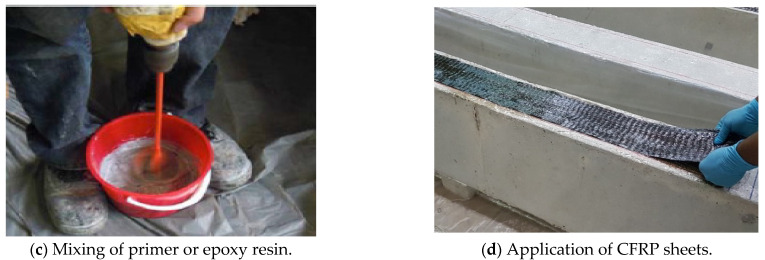
FRP-strengthening procedure for the tested beams.

The strengthening procedure for the tested beams included the preparation of concrete surface, application of wet layup of FRP system, and curing of the impregnating resin. The tension face of the beams was sandblasted and cleaned to ensure proper bond prior to FRP strengthening. Before beginning the strengthening process, FRP sheets were cut to the required length from a roll of CFRP sheets. The CFRP sheets were impregnated with the epoxy adhesive before bonding to the concrete surface. Then, the saturated sheets were placed onto the primed wet surface of concrete and pressed by a small roller that passed over the sheets parallel to the fiber direction until the resin was distributed over the sheets and entrapped air was released. For the second layers of CFRP sheets, the resin was applied to the previously applied layer while it was wet, and a similar laminating procedure as for the previous layer was then followed. The installed wet layup CFRP was then left to cure in laboratory ambient temperature for several days to reach full cure. Figure 3 shows pictures of the FRP-strengthening procedure.

### 2.4. Test Setup and Instrumentation

The beams were simply supported and were tested in four-point bending under a shear span to depth ratio of 2.6, as shown in Figure 2. Electrical resistance strain gauges were used to measure strains developed in the tension steel bars and top concrete surface at midspan of the beams. Strain gauges were also used to measure the strain developed in the steel stirrups in the vicinity of the end of CFRP sheets. The strain developed in CFRP sheets was measured using strain gauges distributed over the length of the sheets. The deflections at midspan and point load locations of the beams were measured using linear variable differential transducers (LVDTs, Tokyo Measuring Instruments Laboratory, Tokyo, Japan.). The load was monotonically applied at a displacement-controlled rate of 1 mm/min using a closed-loop actuator. The applied load, strain readings, and deflection were electronically recorded during the tests using a data acquisition system.

## 3. Test Results and Discussion

A summary of the obtained test results is provided in Table 3. The table gives the applied load and the corresponding midspan deflection at failure. The table also presents the strains at failure in concrete, steel bars, and CFRP sheets, along with the modes of failure of the beams.

### 3.1. Cracking Patterns and Modes of Failure

**Figure 4:**
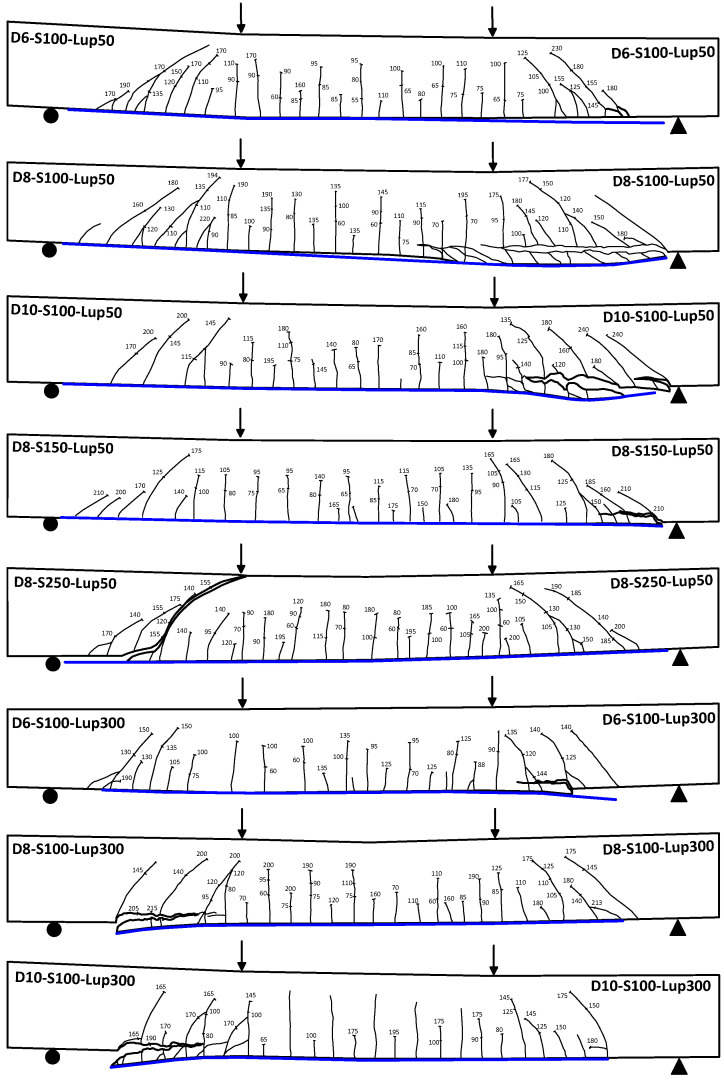

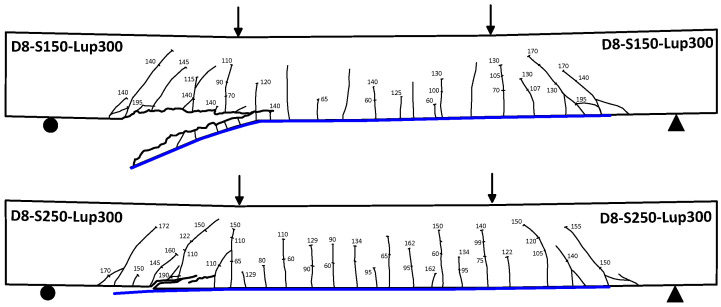
Cracking patterns of the tested beams at failure.

All beams exhibited similar cracking performance at earlier stages of loading. The formation of cracks was initiated in the constant moment zone. The cracks were vertical where the flexural stresses were the highest and the shear stresses were zero. With further loading, additional flexural cracks started to appear in the constant moment zone and in the shear span. The cracks in the shear span became more inclined due to the dominance of shear stresses. Before failure, the shear cracks were prevailing in the vicinity of CFRP plate end region where the plates were terminated in the shear span either close or away from the support. Figure 4 shows the cracking patterns of the tested beams at failure. It can be noticed from the figure that the shear cracks in the plate end regions of the beams of Series 2 became less inclined to the vertical in comparison with the beams of Series 1. This is because the flexural stresses at the plate end region of Series 2 beams were higher than those of Series 1 beams. The increased flexural stresses led to the decrease in the inclination angle of the shear cracks. Thus, as the end of CFRP sheets moved away from the support, the shear cracks at the end region of the sheets became less inclined.

**Figure 5:**
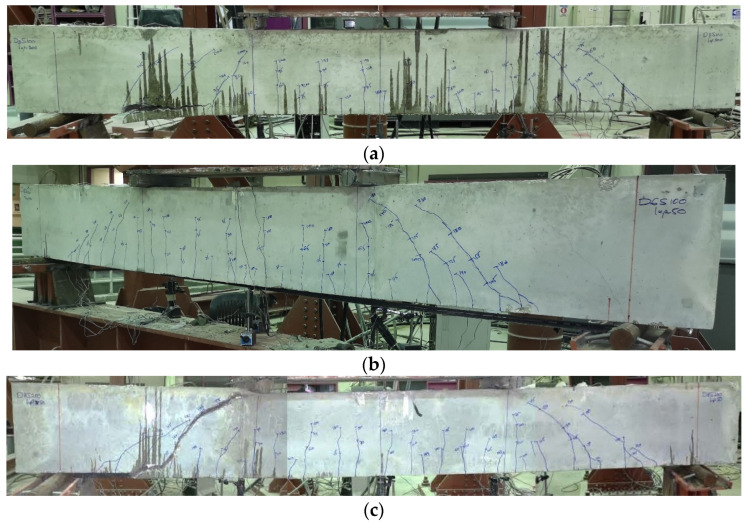
Failure modes: (**a**) CCS (beam D8-S100-L_up_300); (**b**) PEI (beam D6-S100-L_up_50); (**c**) CDC (beam D8-S250-L_up_50).

The typical three PE debonding failures reviewed earlier were encountered in this study. Six of the test beams failed in CCS, three beams failed in PEI debonding, and one beam failed in CDC debonding, as presented in the last column of Table 3. Figure 5 illustrates by photographs these modes of failure. For the beams with CCS, horizontal splitting cracks appeared just before failure at the level of tension steel. The failure occurred at this level by separation of the concrete cover. The concrete cover separated with the CFRP sheets as a one unit without the occurrence of any debonding at the interface between CFRP and concrete substrate. The failure initiated at the plate end and propagated towards the point load. For the beams failed in PEI debonding, the failure also started at the plate end and propagated towards the point load. In this case, the failure occurred at the interface of the CFRP sheets and the concrete substrate. For the beam with CDC debonding, the failure occurred by the formation of a major shear crack that intersected the CFRP sheets near their end. This debonding failure started at the intersection point and propagated to the end of the CFRP sheets along the interface between the sheets and the concrete substrate.

It was interesting to observe that beams with the lowest amounts of shear reinforcement (*ρ_sv_* = 0.2 and 0.28%) in the two series experienced PEI or CDC debonding failures. Beams D6-S100-L_up_50, D6-S100-L_up_300, and D8-S250-L_up_300 failed by PEI debonding, while beam D8-S250-L_up_50 failed by CDC debonding.

### 3.2. Ultimate Capacity

**Figure 6:**
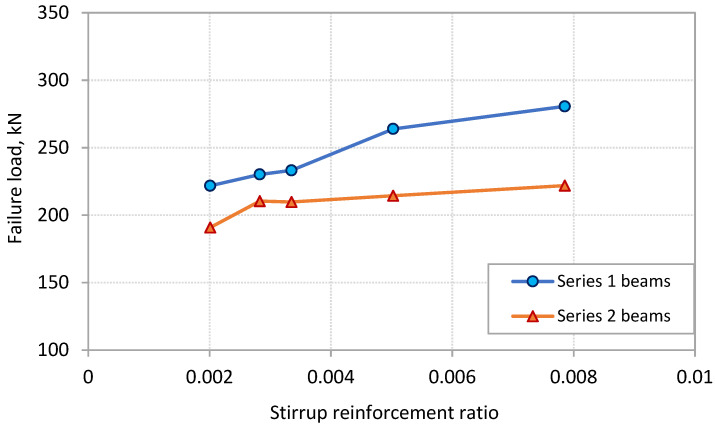
Effect of stirrup reinforcement ratio on PE debonding capacity.

The variation of the failure load against the stirrup reinforcement ratio *ρ_sv_* is plotted in Figure 6 for the beams of the two series tested in this study. The figure indicates that the PE debonding capacity of the beams increased with the increase of *ρ_sv_*. This appears to be more pronounced for the beams of Series 1. Increasing *ρ_sv_* from 0.2% to 0.79% resulted in an increase in the PE debonding capacity of 26.5% for Series 1 beams. On the other hand, beams of Series 2 exhibited an increase of 16.2% in the PE debonding capacity when *ρ_sv_* increased from 0.2% to 0.79%. This result indicates that beams of CFRP sheets extended close to the support obtained more increase in the PE debonding capacity by increasing the stirrup amounts compared with beams with CFRP sheets terminated away from the support. This is attributed to the fact that the efficiency of stirrups in controlling the widening of the shear cracks is affected by the inclination angle of such cracks. The vertical stirrups become more efficient for the cracks with larger inclination angle. As observed in Figure 4 and discussed in the previous subsection, beams of Series 2, where the CFRP sheets terminated in the shear span away from the support, experienced shear cracks with less inclination at the end region of the sheets. This explains why the efficiency of the stirrups in this case was less than that of Series 1 beams with CFRP sheets terminated close to the support. Controlling the widening of the shear cracks at the plate end region reduces the horizontal and vertical components of the relative displacement associated with such cracks. This in turn reduces the interfacial and normal stresses, respectively, at the plate end region, which delays the occurrence of PE debonding and increases the load carrying capacity of the beams.

**Figure 7:**
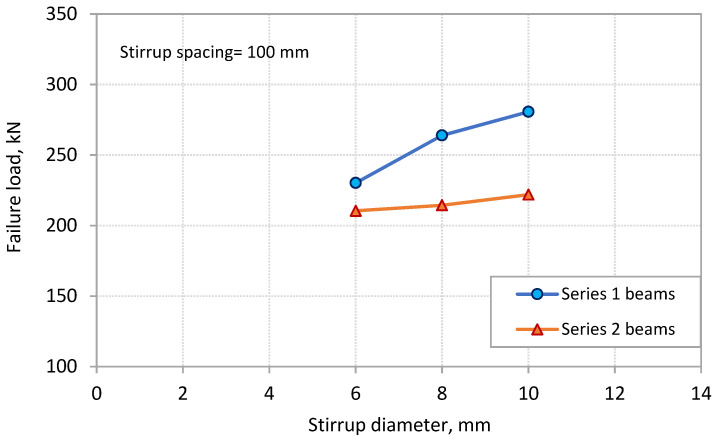
Effect of stirrup diameter on PE debonding capacity.

**Figure 8:**
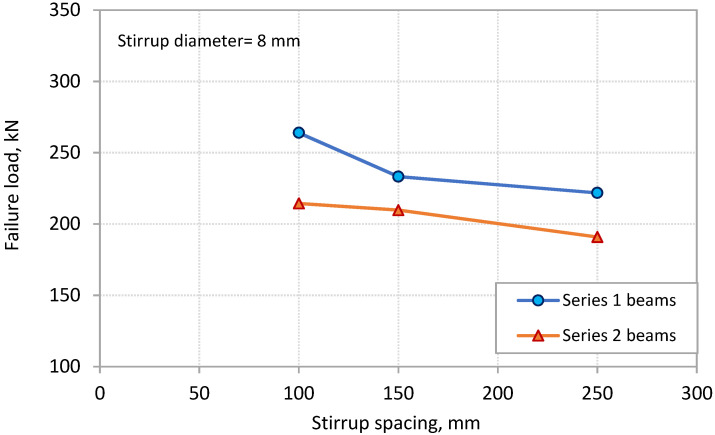
Effect of stirrup spacing on PE debonding capacity.

The effect of the two components of *ρ_sv_*, which are stirrup diameter and spacing, on PE debonding capacity is separated in Figure 7 and Figure 8, respectively. Figure 7 shows the effect of the stirrup diameter on the failure load for the two series of beams. The spacing of stirrups for these beams was kept constant at 100 mm. The figure shows that the failure load increased with the increase in stirrup diameter. The rate of increase was more obvious for beams of Series 1. Increasing the stirrup diameter from 6 mm to 10 mm led to increases of 21.9% and 5.4% in the failure load for the beams of Series 1 and 2, respectively. On the other hand, Figure 8 shows the effect of the spacing of stirrups on the failure load of the beams. The diameter of the stirrups for these beams was kept constant at 8 mm. It can be observed that the failure load increased with the decrease in the stirrup spacing. Decreasing the spacing of stirrups from 250 to 100 mm resulted in increases in the failure load of 19% and 12.3% for the beams of Series 1 and 2, respectively.

### 3.3. Load-Deflection Response

**Figure 9:**
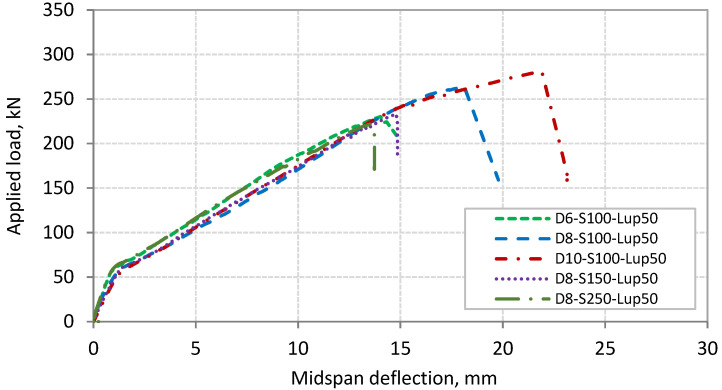
Applied load–deflection relationship for beams of Series 1.

**Figure 10:**
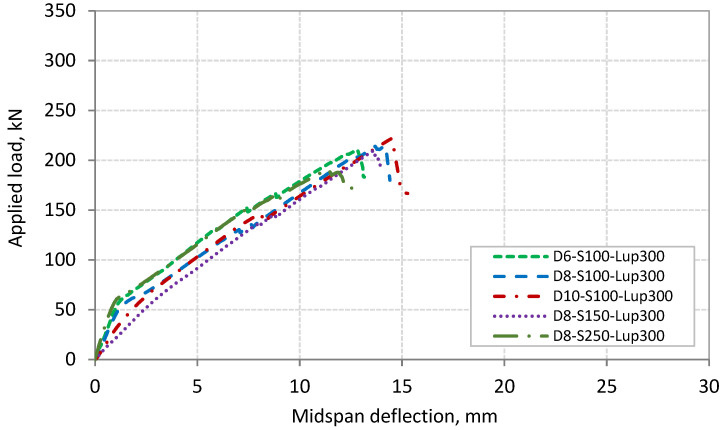
Applied load–deflection relationship for beams of Series 2.

The relationships between the applied load and midspan deflection of Series 1 and 2 beams are shown in Figure 9 and Figure 10, respectively. The two series of beams exhibited similar load–deflection relationship. The figures show that the relationship is bilinear. The first part of this relationship, from the beginning of loading up to the cracking load, represents the behavior of uncracked beam with stiffer performance. The cracking load of the tested beams ranged between 50 and 60 kN. The second part of the relationship, from the cracking load up to failure, represents the postcracking behavior of the beams with lower stiffness compared with the first part. The slope of this part of the relationship appears not to be affected by the onset of shear cracks. The shear cracks started to appear in the shear span of the beams at a load range of 100 to 115 kN. However, the slope of the load–deflection relationship exhibited a slight decrease in beam D8-S250-L_up_50 at a load of 155 kN due to the initiation of the critical diagonal crack. None of the tested beams showed a yielding plateau, where all the beams failed abruptly, indicating the brittle nature of PE debonding. Table 3 also gives the midspan deflection at failure load for each beam. Table 3 and Figure 9 and Figure 10 indicate that the increase in the failure load for the tested beams was associated with the increase in the corresponding midspan deflection.

### 3.4. Strains in Reinforcement and Concrete

Table 3 presents the measured strain values in the concrete and reinforcement at failure. The table shows that the concrete compressive strain measured at the top concrete surface at midspan of the beams, ranged between 1050 and 1620 με for the beams of Series 1. Higher strain values were associated with the beams of higher failure loads and vice versa. The corresponding range for beams of Series 2 was between 860 and 1100 με. These values are significantly lower than the crushing strain of concrete, indicating limited use of the compressive strength of concrete. This is because of the occurrence of PE debonding failure. Beams of Series 1 exhibited larger values than those of Series 2, as they experienced larger failure loads.

The steel strain developed in the main tension steel bars also increased with the increase in failure load, as can be noticed from Table 3. The tested beams developed strains that ranged from 2850 to 4640 με for beams of Series 1 and from 2480 to 2900 με for beams of Series 2. Compared with the yielding strain of 2730 με of the main tension bars, it can be noticed that all beams of Series 1 started yielding before the occurrence of PE debonding failure. On the other hand, beams D6-S100-L_up_300, D8-S150-L_up_300, and D8-S250-L_up_300 of Series 2 failed in PE debonding before the main tension bars yielded. Table 3 shows that the strain developed in the steel stirrups at failure increased with the decrease in *ρ_sv_*. For the beams of Series 1, beam D10-S100-L_up_50 with the largest *ρ_sv_* experienced stirrup strain at failure of 1270 με, compared with 3940 με experienced by beam D8-S250-L_up_50 with the lowest *ρ_sv_*. For the beams of Series 2, beam D10-S100-L_up_300 with the largest *ρ_sv_* gave stirrup strain at failure of 1170 με, compared with 2540 με given by beam D8-S250-L_up_300 with the lowest *ρ_sv_*. Stirrups of beams D6-S100-L_up_50, D8-S150-L_up_50, and D8-S250-L_up_50 of Series 1 and beams D6-S100-L_up_300 and D8-S250-L_up_300 of Series 2 started yielding before failure.

The CFRP sheets showed limited utilization of their strain capacity, as can be seen from the measured strain values given in Table 3. Beams of Series 1 exhibited strain values that ranged from 3170 to 5080 με, whereas beams of Series 2 exhibited a lower range of strain values that ranged from 2800 to 3300 με. These values represent 19.2% to 30.8% and 17% to 20% of the rupture strain of the CFRP sheets used in this study for the beams of the two series, respectively.

## 4. Comparison of Predicted and Experimental PE Debonding Capacity

The PE debonding capacity of the tested beams was predicted using the available prediction models. These models are presented in this section in two categories based on the approach followed for developing the model: shear-based models or fracture energy-based models. Table 4 presents the design equations for predicting PE debonding capacity of FRP-strengthened RC beams for 12 shear-based models. On the other hand, Table 5 presents the design equations for three models that are based on fracture energy approach. The presented models were proposed by researchers or were specified by design codes and guides.

**Figure 11:**
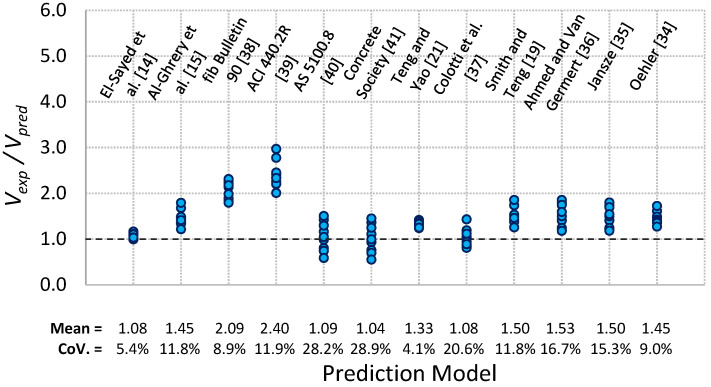
Comparison of experimental and predicted plate end debonding capacity using shear-based models.

**Figure 12:**
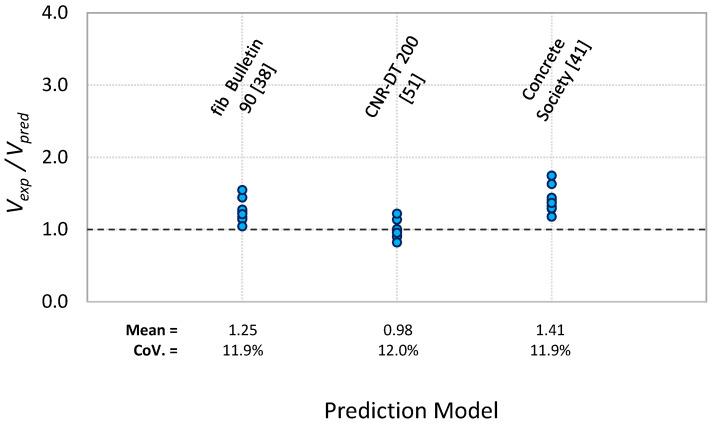
Comparison of experimental and predicted plate end debonding capacity using fracture energy-based models.

The 15 models given in Table 4 and Table 5 were used for predicting PE debonding capacity of the tested beams by determining the shear force *V_pred_* at the PE region at failure. The predicted shear force *V_pred_* was compared with the experimental shear force *V_exp_* at failure for each beam, as given in Table 6 and Table 7 for the two sets of models. Figure 11 and Figure 12 show the ratio of *V_exp_*/*V_pred_* for each prediction model. It can be observed from Table 6 and Figure 11 for the shear-based models that both ACI 440.2R [39] and fib Bulletin 90 [38] methods overly underestimate the PE debonding capacity of the tested beams. This is reflected by the higher value of the average ratio of *V_exp_*/*V_pred_* obtained for the two models, which were 2.4 and 2.09, respectively. The models of Al-Ghrery et al. [15], Teng and Yao [21], Smith and Teng [19], Ahmed and Van Germet [36], Jansze [35], and Oehler [34] exhibited relatively better predictions, but they were still very conservative as the average ratio of *V_exp_*/*V_pred_* for these models ranged between 1.33 and 1.53. The prediction models of AS 5100.8 [40], Concrete Society [41], and Colotti et al. [37] showed average ratios of *V_exp_*/*V_pred_* of 1.09, 1.04, and 1.08, respectively. Although these ratios appear to be more accurate, the models gave inconsistent predictions, as the corresponding coefficients of variation for these ratios were 28.2, 28.9, and 20.6%. Furthermore, each of the three models showed unsafe predictions for 40% of the tested beams. On the other hand, the model proposed by El-Sayed et al. [14] showed better predictions in terms of accuracy and consistency. The average ratio of *V_exp_*/*V_pred_* for this model was 1.08, with a coefficient of variation of 5.4%. For the fracture energy models, Table 7 and Figure 12 indicate that fib Bulletin 90 [38] and Concrete Society [41] methods provided conservative predictions, with average ratios of *V_exp_*/*V_pred_* of 1.25 and 1.41, respectively. It was also indicated that the Italian code CNR-DT 200 [51] provided unsafe predictions for 70% of the tested beams, with an average ratio of *V_exp_*/*V_pred_* of 0.98.

**Figure 13:**
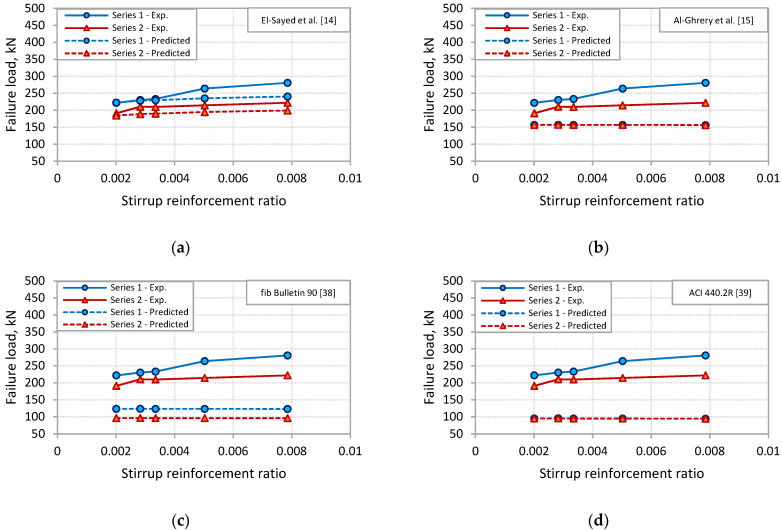

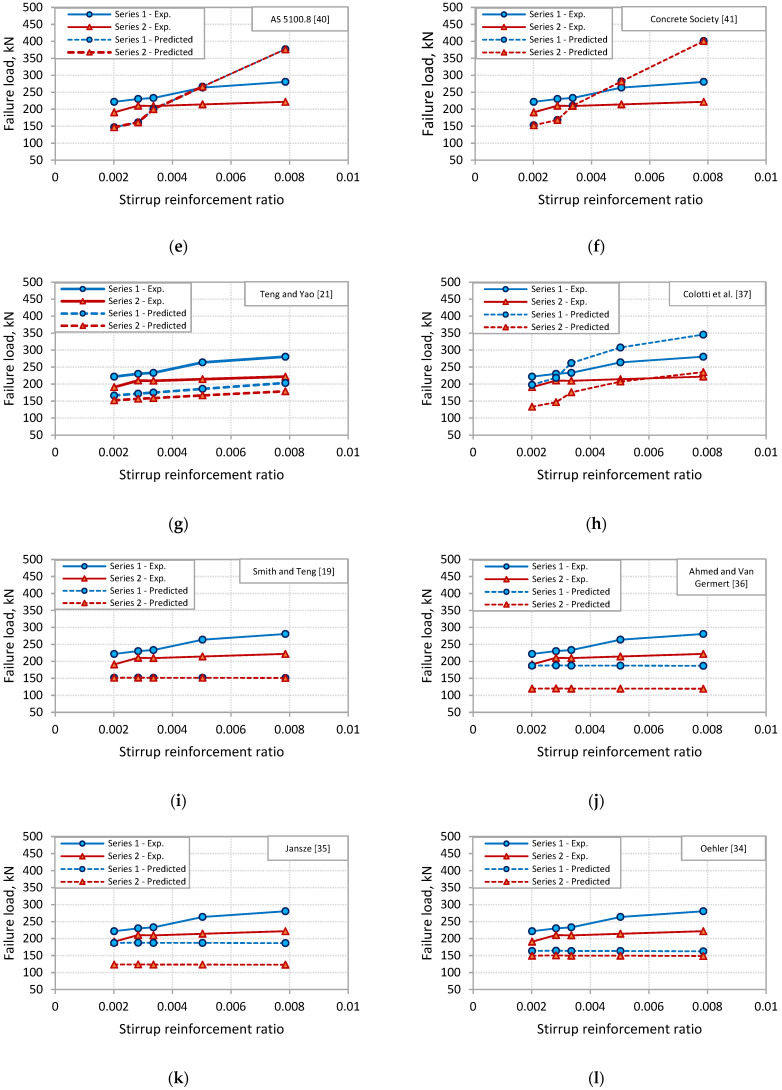
Comparisons of experimental versus predicted values for shear-based models: (**a**) El-Sayed et al. [14]; (**b**) Al-Ghrery et al. [15]; (**c**) fib Bulletin 90 [38]; (**d**) ACI 440.2R [39]; (**e**) AS 5100.8 [40]; (**f**) Concrete Society [41]; (**g**) Teng and Yao [21]; (**h**) Colotti et al. [37]; (**i**) Smith and Teng [19]; (**j**) Ahmed and Van Germert [36]; (**k**) Jansze [35]; (**l**) Oehler [34].

**Figure 14:**
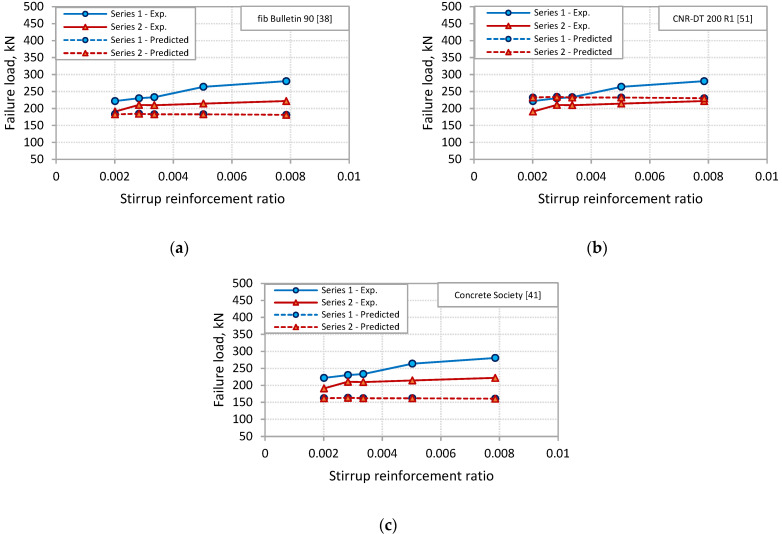
Comparisons of experimental versus predicted values for fracture energy-based code models: (**a**) fib Bulletin 90 [38]; (**b**) CNR-DT 200 R1 [51]; (**c**) Concrete Society [41].

To examine the capability of the predictions models to capture the effect of stirrups on PE debonding capacity of the tested beams, Figure 13 and Figure 14 were plotted for the two sets of models. In these figures, the failure load was plotted against the stirrup reinforcement ratio for experimental and predictions results. For the shear-based models, Figure 13 shows that the models of Al-Ghrery et al. [15], fib Bulletin 90 [38], ACI 440.2R [39], Smith and Teng [19], Ahmed and Van Germet [36], Jansze [35], and Oehler [34] did not reflect the effect of stirrups, as the predicted failure load was constant with varying the stirrup reinforcement ratio for each set of beams. Moreover, the models of Al-Ghrery et al. [15], ACI 440.2R [39], and Smith and Teng [19] did not account for the distance of the termination point of FRP plates to the nearest support, as each of the three models showed the same predicted failure load for the two sets of beams. Figure 13 also indicates that the failure load predicted by AS 5100.8 [40], Concrete Society [41], and Colotti et al. [37] models increased with the increase in stirrup reinforcement ratio. However, models of AS 5100.8 [40] and Concrete Society [41] showed higher rates of increase than the experimental ones, indicating that these models overestimate the effect of stirrups, particularly for beams with larger stirrup reinforcement ratio. It can be also indicated by Figure 13 that both models of El-Sayed et al. [14] and Teng and Yao [21] captured well the effect of stirrups. The predicted failure load by these models appeared to increase with the increase in stirrup reinforcement ratio in a rate approximately comparable to the experimental one. For the fracture energy models, Figure 14 shows that none of the models accounts for the effect of stirrups or the FRP length on PE debonding capacity of the beams. For each model, the predicted failure load was the same for all beams in the two series.

From this comparison, one can notice that the fracture energy-based prediction models did not reflect the effect of stirrups on PE debonding capacity and neither did most of the existing shear-based models. Furthermore, some of the remaining shear-based models highly overestimated the effect of stirrups. Only the models of El-Sayed et al. [14] and Teng and Yao [21] well reflected the effect of stirrups on PE debonding capacity of the tested beams. However, the model of El-Sayed et al. [14] has an advantage over that of Teng and Yao [21], as it provided more accurate predictions for the tested beams.

## 5. Conclusions

The experimental results concerning the effect of stirrups on the PE debonding capacity of FRP-strengthened RC beams were presented. The stirrup amount was varied by varying the stirrup bar diameter and the stirrup spacing. The tests were conducted on beams with CFRP sheets terminated close to the support and on beams with CFRP sheets terminated in the shear span away from the support. The experimental results were compared with the predictions of 15 PE debonding models available in the literature and design codes and guides. The main findings of this study can be summarized as follows:

1. The PE debonding capacity of the beams increased with the increase in the amount of stirrups. Increasing the stirrup bar diameter or decreasing the stirrup spacing was found to increase the failure load of the beams.
2. The effect of stirrups on PE debonding capacity was more pronounced for the beams with CFRP sheets terminated close to the supports when compared with those beams with CFRP sheets terminated away from the supports. This was because the shear cracks in the plate end region were less inclined in the beams with CFRP sheets terminated away from the supports. This reduced the efficiency of stirrups in restricting the opening of the shear cracks in this region, thus reducing the enhancement in PE debonding capacity due to the increase in stirrup amount.
3. The stirrup amount appeared to affect the PE debonding mode of failure. Beams with the lowest amount of stirrups failed in PEI debonding or CDC debonding. Beams with larger amount of stirrups failed in CCS.
4. Most of the existing prediction models did not capture the effect of stirrups on PE debonding capacity of the FRP-strengthened RC beams. Among the 15 prediction models used in this analysis, the models of El-Sayed et al. and Teng and Yao were found to well capture such an effect, with the best predictions provided by El-Sayed et al. model.

## Figures and Tables

**Table 1:** Test beams.

Beam		Stirrup Diameter (mm)	Stirrup Spacing (mm)	Stirrup Reinforcement Ratio, *ρ_sv_* (%)	Cutoff Distance, *L_up_* (mm)
Series 1	D6-S100-L_up_50	6	100	0.28	50
	D8-S100-L_up_50	8	100	0.50	
	D10-S100-L_up_50	10	100	0.79	
	D8-S150-L_up_50	8	150	0.34	
	D8-S250-L_up_50	8	250	0.20	
Series 2	D6-S100-L_up_300	6	100	0.28	300
	D8-S100-L_up_300	8	100	0.50	
	D10-S100-L_up_300	10	100	0.79	
	D8-S150-L_up_300	8	150	0.34	
	D8-S250-L_up_300	8	250	0.20	

**Table 2:** Tensile properties of steel reinforcement and CFRP sheets.

Material	Yield Stress (MPa) (CoV)	Ultimate Tensile Stress (MPa) (CoV)	Elastic Modulus (GPa)	*ε_fu_*
Steel (6 mm)	310 (4.5%)	380 (3.6%)	200	-
Steel (8 mm)	400 (3.8%)	586 (3.5%)	200	-
Steel (10 mm)	500 (1.5%)	574 (2.5%)	200	-
Steel (16 mm)	560 (3.5%)	707 (2.0%)	205	-
Carbon fabric	-	3790	230	0.0165
Adhesive	-	72.4	3.18	0.05

**Table 3:** Summary of test results.

No.	Beam	Failure Load (kN)	Midspan Deflection at Failure (mm)	Strains at Failure (με)				Mode of Failure *
				Concrete	Tension Steel Bars	CFRP	Steel Stirrups	
1	D6-S100-L_up_50	230.2	14	1150	2900	3260	2260	PEI
2	D8-S100-L_up_50	263.9	18.1	1440	3800	4250	1400	CCS
3	D10-S100-L_up_50	280.6	21.8	1620	4640	5080	1270	CCS
4	D8-S150-L_up_50	233.2	14.8	1500	3000	3770	3110	CCS
5	D8-S250-L_up_50	221.8	13.6	1050	2850	3170	3940	CDC
6	D6-S100-L_up_300	210.4	12.6	1020	2700	3120	1830	PEI
7	D8-S100-L_up_300	214.4	14.2	1030	2800	3200	1370	CCS
8	D10-S100-L_up_300	221.9	14.5	1100	2900	3300	1170	CCS
9	D8-S150-L_up_300	209.7	13.6	950	2700	3100	1440	CCS
10	D8-S250-L_up_300	190.9	11.4	860	2480	2800	2540	PEI

* CCS = Concrete cover separation; CDC = Critical diagonal crack debonding; PEI = Plate end interfacial debonding.

**Table 4:** Shear-based models for prediction of plate end debonding.

Reference	Debonding Criteria (Units: N and mm)
El-Sayed et al. [14]	$$V_{db,end}=2.17\beta_{v}\beta_{L}{\left(f_{c}^{\prime }\rho_{eq}\frac{d_{eq}}{a}\right)}^{1/3}bd_{eq}$$ where $V_{db,end}$ is the shear force applied at the plate end at failure, *f’_c_* is the specified concrete compressive strength of the beam, *ρ_s_* is the longitudinal reinforcement ratio of the main steel, *a* is the shear span. Additionally, the equivalent effective depth *d_eq_* and equivalent reinforcement ratio *ρ_eq_* are as given by $$d_{eq}=\frac{A_{s}E_{s}d_{s}+A_{f}E_{f}h}{A_{s}E_{s}+A_{f}E_{f}}\;,\;\rho_{eq}=\left(A_{s}+A_{f}\frac{E_{f}}{E_{s}}\right)/\left(bd_{eq}\right)$$ where $A_{s}$ and $E_{s}$ are the internal steel area and modulus of elasticity; $A_{f}$ and $E_{f}$ are the external FRP area and modulus of elasticity; b, *d_s_*, and h are beam width, effective depth of tension steel, and total depth of beam, respectively. The factors $\beta_{v}$ and $\beta_{L}$ account for the influence of the shear reinforcement and the location of FRP cutoff point, respectively, and are given by $$\beta_{v}=2.15{\left(\rho_{v}\right)}^{0.06}\;,\;\beta_{L}=0.57{\left(\frac{l_{up}}{a}\right)}^{-0.34}\;\leq 1.0$$ in which $l_{up}$ represents the unplated length, and $\rho_{v}$ is the shear reinforcement ratio.
Al-Ghrery et al. [15]	$$V_{CCS}=46\alpha_{1}e^{\alpha_{2}}e^{\alpha_{3}}\ln f_{c}^{\prime }$$ where $$\alpha_{1}=\sqrt{\frac{d_{s}^{2/3}V_{uc}}{f_{c}^{\prime }}}\;,\;\alpha_{2}=\sqrt{\frac{b^{2/3}A_{s}}{V_{uc}}}\;,\;\alpha_{3}=\frac{7.11A_{s}}{V_{uc}}+\frac{f_{c}^{\prime }}{2V_{us}}$$ where $V_{CCS}$ is the shear force to initiate CCS failure, $f_{c}^{\prime }$ is the mean compressive cylinder concrete strength, $V_{us}$ is the steel contribution to shear capacity, and $V_{uc}$ is concrete contribution to shear strength as per AS 5100.5 [45].
fib Bulletin 90 [38]	$$V_{RD,c,fe}=0.75\left[1+19.6\frac{{\left(100\rho_{s}\right)}^{0.15}}{l_{up}}\right]V_{Rd,c}$$ $V_{RD,c}=\left[C_{RD,c}k{\left(100\rho_{s}f_{c}^{\prime }\right)}^{1/3}\right]bd_{s}$ with a minimum of: $V_{RD,c}=\left[0.035k^{1.5}f_{c}^{'}{}^{0.5}\right]bd_{s}$ $$k=1+\sqrt{\frac{200}{d_{s}}}\leq 2\;,\;C_{RD,c}=\frac{0.18}{\gamma_{c}}$$ where $V_{RD,c,fe}$ is the shear force required to initiate CCS, $V_{RD,c}$ is the shear capacity for members not requiring shear reinforcement following EN 1992-1-1 [46], and $\gamma_{c}$ is a partial factor for concrete.
ACI 440.2R [39]	An upper limit for the factored shear force at the termination point of the plate was provided to avoid PE debonding: $$V_{db,end}<0.67V_{c}$$ where $V_{c}$ is the concrete shear strength of the beam section determined in accordance with the ACI 318 Code [47].
AS 5100.8 [40]	An upper limit for the acting shear force at the plate end region was suggested by $$V_{db,end}<0.67V_{u}$$ where $V_{u}$ is the nominal shear strength of the beam section determined in accordance with the Australian Standard AS 5100.5 [45].
Concrete Society [41]	An upper limit for the acting shear force at the plate end region is suggested to avoid PE debonding: $$V_{db,end}<0.67V_{Rd}$$ where $V_{Rd}$ is the shear strength of the beam section determined in accordance with Section 6.2 of Eurocode 2 [46].
Teng and Yao [21]	The flexural debonding moment of FRP plate end located in a pure bending region is $$M_{db,f}=0.488M_{u,o}/{\left(\alpha_{flex}\;\alpha_{axial}\;\alpha_{w}\right)}^{1/9}\leq M_{u,o},$$ where $\alpha_{flex}$, $\alpha_{axial}$ and $\alpha_{w}$ are parameters defined by $$\alpha_{flex}={\left(EI\right)}_{f,c}-{\left(EI\right)}_{o,c}/{\left(EI\right)}_{o,c}\;,\;\alpha_{axial}=E_{f}t_{f}/E_{c}d_{s}\;,\;\alpha_{w}=b/b_{f}\leq 3,$$ where ${\left(EI\right)}_{f,c}$ and ${\left(EI\right)}_{o,c}$ are the flexural rigidities of the cracked section with and without EB FRP, respectively, and $M_{u,0}$ is the theoretical ultimate moment of the un-plated section, which is also the upper bound of the flexural debonding moment $M_{db,f}$. The debonding shear force at an FRP plate end located in a region of (nearly) zero moment is $$V_{db,s}=V_{c}+V_{f}+\varepsilon_{v,e}\bar{V_{s}},$$ where $\bar{V_{s}}$ is the shear force carried by the shear steel reinforcement per unit strain, given by $$\bar{V_{s}}=A_{sv}E_{sv}d_{e}/s_{v}$$ The effective strain in the shear steel reinforcement, $\varepsilon_{v,e}$ , is given by (in *µε*) $$\varepsilon_{v,e}=10/{\left(\alpha_{flex}\;\alpha_{E}\;\alpha_{t}\;\alpha_{w}\right)}^{1/2},\;\text{with}\;\alpha_{E}=E_{f}/E_{c};\;\alpha_{t}={\left(t_{f}/d_{s}\right)}^{1.3},$$ For the predictions of the shear capacity contributed by the concrete and FRP plate $\left(V_{c}+V_{f}\right)$, the authors proposed that prestress model of Oehlers et al. [48,49] be adopted. An interaction between plate end shear and bending was proposed as follows: $${\left(M_{db,end}/0.85M_{db,f}\right)}^{2}+{\left(V_{db,end}/0.85V_{db,s}\right)}^{2}=1.0$$
Colotti et al. [37]	The ultimate shear load for the plate end failure mode of FRP-strengthened beam is $$V_{db,end}=p_{y}d_{s}\left[\phi +\alpha -\sqrt{{\left(\phi +\alpha \right)}^{2}-2\phi \beta }\right]\;,\;\;\;p_{y}>0,$$ with $p_{y}=A_{v}f_{yv}/s_{v}$; $\alpha =a/d_{s}$; $\;\beta =l_{a}/d_{s}$; $\phi =U_{y}/p_{y}$ The limiting bond strength, $U_{y}$, is given by $$U_{y}=\{\begin{array}{c} b_{m}\left[2.77+0.06\left(f_{c}^{\prime }-20\right)\right]\;\;\;\;\;\;\;\;\;\;\;\;\;\;\;\;\;\;\mathrm{for}\;\mathrm{PE}\;\mathrm{debonding}\\ \mathrm{Min}\left\{b_{m}\left[2.77+0.06\left(f_{c}^{\prime }-20\right)\right],\;\left(f_{ct}s_{c}b/C_{c}\right)\right\}\;\;\;\;\;\;\;\;\;\;\;\mathrm{for}\;\mathrm{CCS} \end{array}$$ where $b_{m}=\left(b+b_{f}\right)/2$; $s_{c}$ is the width of the tie element, given by $s_{c}=l_{c}/5$, in which the crack spacing, $l_{c}$, could be calculated according to Eurocode2 by $l_{c}=50+0.25k_{1}k_{2}\phi_{s}/\rho_{r}$, where $k_{1}$ and $k_{2}$ equal to 0.8 and 0.5, respectively; and $\rho_{r}=A_{se}/2.5bc_{c}$, in which $A_{se}=A_{s}/2$.
Smith and Teng [19]	The debonding shear force at the plate end, $V_{db,end}$, is given by $$V_{db,end}=\eta V_{c},$$ where $V_{c}$ is the shear capacity of the concrete beam alone without the contribution from the shear reinforcement following the AS 3600 [50], given by $$V_{c}=\left[1.4-\left(\frac{d_{s}}{2000}\right)\right]bd_{s}{\left[\rho_{s}f_{c}^{\prime }\right]}^{1/3}$$ The factor η is taken equal to 1.5.
Ahmed and Van Germert [36]	The critical shear force at the FRP plate end that causes debonding, $V_{db,end}$, is given as a function of the shearing stress, $\tau_{PES}$, by $$V_{db,end}=\left(\;\tau_{PES}+{\Delta \tau }_{mod}\right)\;bd_{s}$$ in which $${\Delta \tau }_{mod}=\tau_{PES}bd_{s}\left(\frac{S_{s}}{I_{s,c}b_{f}}-\frac{S_{f}}{I_{f,c}b_{a}}\right)+6188.5\left(\frac{\tau -4.121}{bd_{s}}\right)$$ $$\tau =\left(0.15776\sqrt{f_{c}^{\prime }}+\frac{17.2336\rho_{s}d_{s}}{a}\right)+0.9\frac{A_{sv}f_{yv}}{s.b}$$ where $\tau_{PES}$ is the same as suggested by Jansze [35]. $S_{f}$ and $S_{s}$ are first moments of area about the neural axis for FRP plate and that of an equivalent steel plate, respectively. The equivalent steel plate is the one that has the same tensile capacity and width as that of the FRP plate but with an equivalent thickness determined, assuming that the yield stress of steel is 550 MPa. The terms $I_{f,c}$ and $I_{s,c}$ are the moments of inertia of a cracked plated section with FRP plates and equivalent steel plates, respectively. $b_{f}$ and $b_{a}$ are the widths of FRP plate and adhesive, respectively. The terms $A_{sv}$ and $f_{yv}$ are the cross sectional area and yield stress of the steel stirrups, respectively, whereas s is the stirrup spacing.
Jansze [35]	The critical shear force at the FRP plate end that causes debonding, $V_{db,end}$, is given as a function of the shearing stress, $\tau_{PES}$, by $$V_{db,end}=\tau_{PES}bd_{s},\;\text{with}\;\tau_{PES}=0.18\;\sqrt[{3}]{3d_{s}/a_{v}^{\prime }}\left(1+\sqrt{200/d_{s}}\right)\sqrt[{3}]{100\rho_{s}f_{c}^{\prime }},$$ where $a_{v}^{\prime }$ is a modified shear span, equal to $$a_{v}^{\prime }=\sqrt[{4}]{{\left(1-\sqrt{\rho_{s}\;}\right)}^{2}d_{s}l_{up}{}^{3}/\rho_{s}}$$ If $a_{v}^{\prime }$ is greater than the actual shear span, a, then the value $\left(a_{v}^{\prime }+a\right)/2$ should be used.
Oehler [34]	$$\frac{M_{db,end}}{M_{db,f}}+\frac{V_{db,end}}{V_{db,s}}\leq 1.17\;\text{and}\;M_{db,end}\leq M_{db,f},\;V_{db,end}\leq V_{db,s}$$ where $M_{db,end}$ and $V_{db,end}$ are the bending moment and shear force applied at the plate end at failure, respectively, $M_{db,f}$ is the debonding moment at the end of a plate terminated in the constant moment region, and $V_{db,s}$ is the debonding shear force at the end of a plate terminated near the support. The ultimate debonding moment $M_{db,f}$ is given by the following equation: $$M_{db,f}=\frac{E_{c}\;I_{tr,c}\;f_{ct}}{0.9\;E_{f}\;t_{f}}$$ where $E_{c}$ and $E_{f}$ are the moduli of elasticity of the concrete and the FRP, respectively, $I_{tr,c}$ is the cracked second moment of area of the plated section transformed into equivalent concrete, $f_{ct}$ is the cylinder splitting tensile strength of concrete, and $t_{f}$ is the FRP plate thickness.

**Table 5:** Fracture energy-based models given by codes for the prediction of plate end debonding.

Reference	Debonding Criteria (Units: N and mm)
fib Bulletin 90 [38]	$$\varepsilon_{fd}=\{\begin{array}{c} k_{m}k_{b}\sqrt{\frac{2f_{cm}^{2/3}}{n_{f}t_{f}E_{f}}}\;\;\;\;\;\;\;\;\;\;\;\;\;\;\;\;\;\;\;\;\;\;\;\;\;\;\;\;\;\;\;\;\;,l_{b}\;l_{e}\\ k_{m}k_{b}\sqrt{\frac{2f_{cm}^{2/3}}{n_{f}t_{f}E_{f}}}.\left(\;l_{b}/l_{e}\right)\left(2-l_{b}/l_{e}\right)\;\;\;\;\;\;\;,l_{b}<l_{e} \end{array}$$ $$k_{m}=0.25\;,\;k_{b}=\sqrt{\left(2-b_{f}/b\right)/\left(1+b_{f}/b\right)}$$ The effective bond length, $l_{e}$, is calculated by $$l_{e}=\frac{\pi }{k_{b}}\sqrt{n_{f}t_{f}E_{f}/8f_{cm}^{2/3}}$$
CNR DT200 [51]	$$\varepsilon_{fd}=\{\begin{array}{c} 1/\gamma_{f,d}\sqrt{2\Gamma_{Fd}/n_{f}t_{f}E_{f}}\;\;\;\;\;\;\;\;\;\;\;\;\;\;\;\;\;\;\;\;\;\;\;\;\;\;\;\;\;\;\;\;,l_{b}\;l_{e}\\ 1/\gamma_{f,d}\sqrt{2\Gamma_{Fd}/n_{f}t_{f}E_{f}}.\left(l_{b}/l_{e}\right)\left(2-l_{b}/l_{e}\right)\;\;\;\;\;\;,l_{b}<l_{e} \end{array}$$ where $\gamma_{f,d}$ is a partial factor (1.2‒1.5); $\Gamma_{Fd}$ is the design value of the specific fracture energy of the FRP-concrete interface, given by $$\Gamma_{Fd}=k_{b}k_{G}\sqrt{f_{c}^{\prime }\;f_{ct}}$$ in which $k_{G}$ is a corrective factor taken for pre-cured FRP (0.063 mm for the mean value and 0.023 mm for the 5% fractile value) and for wet lay-up FRP (0.077 mm for the mean value and 0.037 mm for the 5% fractile value); $k_{b}$ is a geometric coefficient given by $$k_{b}=\{\begin{array}{c} \sqrt{\left(2-b_{f}/b\right)/\left(1+b_{f}/b\right)}\geq 1,\;\;\;\;\;b_{f}/b\geq 0.25\\ \;\;\;\;\;\;\;\;\;\;\;\;\;\;\;\;\;\;\;\;\;\;\;\;\;\;\;\;\;\;\;\;\;\;\;\;\;\;\;\;\;\;1.18,\;\;\;\;\;b_{f}/b<0.25 \end{array}$$ The effective bond length, $l_{e}$ , is calculated by $$l_{e}=min\left\{\left(1/\gamma_{Rd}f_{bd}\right)\sqrt{\pi^{2}n_{f}t_{f}E_{f}\Gamma_{Fd}/2},\;200\;mm\right\}$$ where $f_{bd}=2\Gamma_{Fd}/S_{u}$, with $s_{u}=0.25\;\mathrm{mm}$, and $\gamma_{Rd}=1.25$ as a corrective factor.
Concrete Society TR55 [41]	$$\varepsilon_{fd}=\{\begin{array}{c} 0.5k_{b}\sqrt{f_{ct}/n_{f}t_{f}E_{f}}\;\;\;\;\;\;\;\;\;\;\;\;\;\;\;\;\;\;\;\;\;\;\;\;\;\;\;\;\;\;\;\;\;\;\;\;\;\;\;\;\;\;,l_{b}\;l_{e}\\ 0.5k_{b}\sqrt{f_{ct}/n_{f}t_{f}E_{f}}.\;l_{b}/l_{b,\;max}\;\left(2-l_{b}/\;l_{b,\;max}\right)\;,l_{b}<l_{e} \end{array}$$ where *k_b_* is a geometry factor given by $$k_{b}=1.06\sqrt{\left(2-b_{f}/b\right)/\left(1+b_{f}/400\right)}\geq 1,\;\text{with}\;b_{f}/b\geq 0.33$$ The maximum anchorage length, $l_{e}$, is given by $l_{e}=0.7\sqrt{n_{f}t_{f}E_{f}/f_{ct}}$

**Table 6:** Comparisons of shear-based models for prediction of plate end debonding capacity of the tested beams.

No.	*V_exp_* (kN)	Experimental to Predicted Shear Load at Failure, *V_exp_/V_pred_*											
		El-Sayed et al. [14]	Al-Ghrery et al. [15]	Fib Bulletin 90 [38]	ACI 440. 2R [39]	AS 5100.8 [40]	Concrete Society [41]	Teng and Yao [21]	Colotti et al. [37]	Smith and Teng [19]	Ahmed and Van Germert [36]	Jansze [35]	Oehler [34]
1	115.1	1.01	1.46	1.86	2.41	1.43	1.37	1.34	1.05	1.51	1.22	1.22	1.40
2	132.0	1.12	1.68	2.14	2.78	0.99	0.94	1.42	0.86	1.74	1.41	1.41	1.61
3	140.3	1.17	1.79	2.28	2.97	0.74	0.70	1.38	0.81	1.86	1.50	1.50	1.73
4	116.6	1.02	1.49	1.89	2.46	1.16	1.11	1.33	0.89	1.54	1.24	1.24	1.43
5	110.9	1.00	1.41	1.80	2.34	1.51	1.45	1.33	1.12	1.46	1.18	1.18	1.36
6	105.2	1.11	1.34	2.18	2.20	1.30	1.25	1.34	1.43	1.38	1.75	1.70	1.40
7	107.2	1.10	1.37	2.23	2.26	0.80	0.76	1.29	1.03	1.41	1.79	1.74	1.43
8	111.0	1.11	1.42	2.31	2.35	0.59	0.55	1.24	0.94	1.47	1.86	1.80	1.49
9	104.9	1.10	1.34	2.18	2.21	1.05	1.00	1.32	1.19	1.38	1.75	1.70	1.40
10	95.5	1.04	1.22	1.99	2.01	1.30	1.25	1.25	1.43	1.26	1.59	1.55	1.27
Mean=		1.08	1.45	2.09	2.40	1.09	1.04	1.33	1.08	1.50	1.53	1.50	1.45
CoV.=		5.4%	11.8%	8.9%	11.9%	28.2%	28.9%	4.1%	20.6%	11.8%	16.7%	15.3%	9.0%

**Table 7:** Comparisons of fracture energy-based models given by codes for the prediction of plate end debonding capacity of the tested beams.

No.	*V_exp_* (kN)	Experimental to Predicted Shear Load at Failure, *V_exp_/V_pred_*		
		Fib Bulletin 90 [38]	CNR-DT 200 [51]	Concrete Society [41]
1	115.1	1.25	0.98	1.41
2	132.0	1.44	1.14	1.63
3	140.3	1.55	1.22	1.75
4	116.6	1.28	1.00	1.44
5	110.9	1.21	0.96	1.37
6	105.2	1.14	0.90	1.29
7	107.2	1.17	0.92	1.32
8	111.0	1.22	0.96	1.38
9	104.9	1.15	0.90	1.29
10	95.5	1.04	0.82	1.18
Mean=		1.25	0.98	1.41
CoV.=		11.9%	12.0%	11.9%

## Data Availability

The data presented in this study are available on request from the corresponding author. The data are not publicly available due to privacy issues.
